# Determinants of life expectancy in high longevity countries: evidence from machine learning

**DOI:** 10.3389/fpubh.2025.1687871

**Published:** 2025-12-02

**Authors:** Hong Zhang, Haixiang Qiao, Xiuping Li, Ijaz Uddin, Ruitao Li, Xiaolan Zhang

**Affiliations:** 1 School of Finance and Economics, Qingdao Binhai University, Qingdao, China; 2 Department of Economics, Abdul Wali Khan University Mardan, Mardan, Pakistan; 3 Technology, Education, and Management at the Graduate School of Business & Advanced Technology Management, Assumption University of Thailand, Bangkok, Thailand; 4 Intelligent Transportation School, Yunnan Vocational College of Transportation, Kunming, China; 5 The First Affiliated Hospital of Zhejiang Chinese Medical University (Zhejiang Provincial Hospital of Chinese Medicine), Hangzhou, China

**Keywords:** economic, social, institutional, life expectancy, machine leaning

## Abstract

Health is directly aligned with Sustainable Development Goal (SDG) 3: Good Health and Well-Being, which emphasizes ensuring healthy lives and promoting well-being for all at all ages. The present study investigates the determinants of life expectancy (LEX) by incorporating a comprehensive set of factors: CO₂ emissions as an environmental factor; GDP, health expenditure, and research and development (R&D) as economic factors; education and individual internet use as social factors; and rule of law and government effectiveness as institutional factors. Using panel data for the top 20 high-life-expectancy countries covering the period 2001–2023, this study applies both traditional econometric techniques namely, PMG, fixed effects, and FMOLS estimators and advanced machine learning approaches, specifically Gradient Boosting and Random Forest. The regression results reveal that CO₂ emissions negatively affect LEX, whereas GDP, health expenditure, education, internet use, rule of law, government effectiveness, and R&D exert positive influences. The machine learning results further indicate that GDP, health expenditure, and education are the three most critical predictors of LEX in both Gradient Boosting and Random Forest models, with GDP emerging as the most dominant factor. Institutional variables such as rule of law, government effectiveness, and R&D display moderate importance, while CO₂ emissions and individual internet use consistently rank as the least influential. In terms of predictive performance, Gradient Boosting outperforms Random Forest across evaluation metrics, demonstrating lower errors and higher explanatory power. In light of these findings, this study also provides important policy implications to enhance LEX.

## 1 Introduction

Life expectancy (LEX) refers to the average number of years a newborn is expected to live, assuming current mortality patterns remain constant throughout their life. It is a widely used indicator of population health and development. LEX reflects both healthcare access and broader socio-economic conditions influencing survival. Economic growth influences LEX and health, especially in developing and impoverished countries. A stable and strong economy frequently results in better public health programs, higher living standards, and better healthcare infrastructure all of which raise life expectancy (1). Increased per capita income reflects a country’s investment in social indicators such as health, education, pensions, food facilities, sanitation, and environmental status (2, 3). The countries with higher GDP per capita tend to have higher living standards, better healthcare facilities, and more public investment in medical services, all of which raise life expectancy. Those who earn more money typically have easier access to wholesome food, potable water, and high-quality healthcare, which lowers mortality rates (4, 5). A growing economy allows for investments in healthcare infrastructure, education, and social services, which improve life expectancy. However, the distributional elements of economic growth, wealth inequality, and discrepancies in access to healthcare services can bring complexity that affects different parts of the population differently (6, 7). Increasing wealth only enhances happiness until fundamental necessities are covered. After that, income can lead to hunger relief and sickness prevention for children (8). As a result, much of the improvement in people’s happiness stemmed from the reduction of child and infant mortality; millions of children were destroyed because of abject poverty and the failure to implement basic improvements in sanitation and public health (9, 10).

Environmentalists and policymakers are concerned about air pollution, particularly carbon emissions (CO2E) from industrialization and energy consumption. These emissions have been identified as a contributing factor to global health issues (11, 12). Carbon emissions from using fossil fuels have caused negative externalities globally. Energy usage, particularly from fossil fuels, as well as manufacturing and construction activities, has a severe impact on the environment, leading to deterioration, poor health outcomes, and shortened life expectancy, specifically for aquatic life (13–15). A World Health Organization (16) report states that air pollution kills around seven million individuals every year (17). According to Hill et al. (18), air pollution is an environmental issue that contributes to mortality in all societal sectors. Air pollution presents serious health hazards to the earth, significantly increasing the rate of premature death and the development of pollution-related illnesses around the world (19, 20). Changes in climatic patterns brought on by CO2 emissions can impact the distribution and prevalence of disease vectors, which in turn can impact the occurrence of vector-borne illnesses like dengue fever and malaria. This emphasizes how complex and multidimensional the relationship is between life expectancy and CO2 emissions (21). According to Das and Debanth (22), CO2 emissions significantly affect life expectancy via a wide range of interrelated routes. Fossil fuel combustion generates pollutants that significantly increase air pollution and are acknowledged as a major contributor to CO2 emissions (23). Environmental factors cause 33% of diseases and 13 million deaths each year. Over 3 billion people use dirty fuel, and air pollution is responsible for 7 million of these deaths—90 percent of the world’s population breathes filthy air. Poor environmental management causes 2 million fatalities each year owing to water-related problems, infectious diseases, and other negative consequences (24). Transportation is the primary source of CO2 emissions, which also contribute to climate change and present several health hazards, such as cardiovascular and pulmonary conditions, as well as some forms of cancer (25, 26).

Spending on health care is beneficial when it is adequate. On the other hand, poor healthcare spending may make it more difficult for the underprivileged to get necessary medical treatment (27). The World Health Organization defines health expenditure as both capital investments in healthcare infrastructure and consumption of products and services to improve health outcomes (28, 29). According to the OECD, a 10% increase in per capita health expenditure results in a 3.5-month increase in life expectancy, implying that health spending has been the dominant driver of longevity gains in recent decades, particularly among older persons (30–32). Health outcomes have significantly improved globally during the last few decades. This has happened at the same time that health expenditure has increased. Between 1990 and 2013, life expectancy at birth rose from 64 to 71 years, the maternal mortality ratio dropped by 45%, and the under-five mortality rate dropped by 49% globally (33, 111). Short-term healthcare facilities, such as doctor and hospital bed availability, have an impact on life expectancy. On the other hand, long-term improvements may necessitate ongoing expenditures in health technology and infrastructure (34–36). Health infrastructure can either directly protect health (e.g., public sanitation systems) or support other health-promoting activities (37). Investing in the health system strengthens its infrastructure and improves individual health situations (38, 39). Increasing the health care budget improves accessibility and lowers costs, reducing the likelihood of mortality (40). Health indicators like infant mortality and total mortality have been significantly impacted by the pandemic’s worsening of the issues that healthcare workers experience, including workload, mental health, and work-life balance (41, 42). For the healthcare system to remain sustainable, it is essential to improve working conditions, increase training, decrease workload, and strengthen the health staff (43, 44).

ICT increases life expectancy by making health information easier to obtain and sharing knowledge about epidemics, good nutrition, and health (45, 46). ICT enhances patient-physician communication and health awareness, which can boost early disease identification and treatment and help people make better decisions about their quality of life (47, 48). Advances in ICT have a significant socioeconomic link to health outcomes. However, the greatest advantage of aging and lifespan is the creation of chances for individuals to interact, with ICT spending functioning as a policy tool for governments (49). The World Health Organization (WHO) defines “eHealth as the use of (ICT) services in the health sector, such as e-health, m-health, and telemedicine, as part of the extensive use of digital technology for health activities in many contexts (50, 51). By making it easier to gather, organize, and disseminate health information promptly, ICT elements such as accessibility, affordability, and applications may make it possible to provide effective public health (52, 53). ICTs give the infrastructure and resources to construct large-scale, population-level applications such as health information networks, surveillance systems, and telemedicine (54–56).

People with high levels of education, use their information, knowledge, and life experiences to avoid risk factors for health problems and to adopt healthy habits, including quitting smoking, abstaining from alcohol, and exercising frequently (57, 58). According to Mirowsky and Ross (59), more education leads to steady and well-paid jobs and increased income, which can cover expenses such as nutritious food, better housing, and high-quality medical care. Education delivers socio-psychological resources that, through instrumental and emotional support, can promote lifespan and good health (60). Another factor that influences the association between education and health is the fact that those with higher levels of education are more likely to get married and stay married (61, 62). Education has an indirect effect on life expectancy and health by raising the risk of non-fatal work-related accidents and affecting employment in occupational classes that pay more and have access to health insurance. This relationship is complex to break, but it will probably continue (63, 64). Health needs are hampered by un insurance, yet greater wages decrease unmet medical demands. Through societal impacts, more wealth not only meets biological demands but also lengthens life expectancy by empowering people to take charge of their own lives and meet their biological needs. This emphasizes how crucial cash is for meeting medical demands (65–68). Education also affects life expectancy by influencing economic growth and development, while it is a significant factor in determining human capital, which in turn influences economic growth and development (69–71).

**Figure 1:**
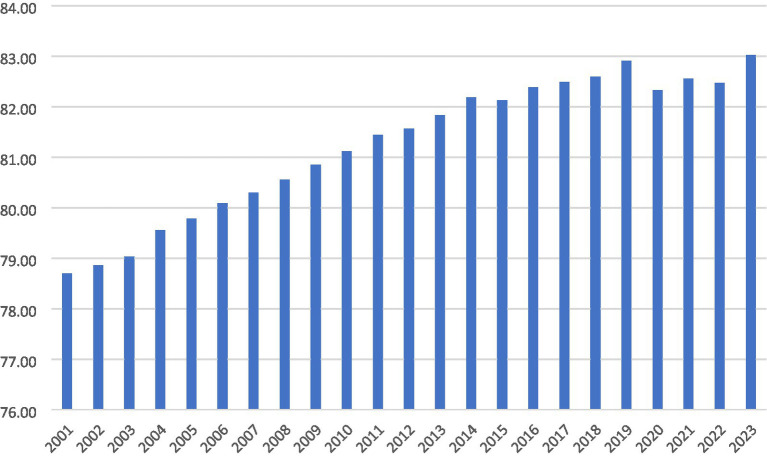
Trends of life expectancy in selected countries.

In Figure 1 shows the data of the average LEX at birth for the selected countries (Belgium, Canada, France, Iceland, Ireland, Israel, Italy, Japan, Korea, Luxembourg, Malta, Netherlands, New Zealand, Norway, Portugal, Qatar, Singapore, Spain, Sweden, and Switzerland) over the period 2001–2023. In 2001, the average LEX of the top 20 countries stood at 78.7 years. It showed a steady upward trend, crossing 81 years by 2010. This growth reflects improvements in healthcare, nutrition, and living standards. From 2014 to 2019, LEX stabilized above 82 years, peaking at 82.91 in 2019. In 2020, it dropped to 82.33 years, mainly due to the COVID-19 pandemic. Subsequently, recovery followed, with LEX reaching 83.02 years in 2023. Overall, LEX improved by 4.3 years over 2001–2023, despite temporary shocks. Countries such as Belgium, Canada, France, Iceland, Ireland, Israel, Italy, Japan, Luxembourg, Netherlands, New Zealand, Norway, Singapore, Spain, Sweden, and Switzerland are among the world’s richest economies. In these nations, people tend to live longer, which encourages both public and private sectors to invest more in health and education. Longer LEX increases the demand for quality healthcare and lifelong learning. Governments also allocate larger budgets to sustain a healthy, skilled, and productive population. As a result, wealth and longevity reinforce each other, creating a cycle of continuous investment in human capital.

To the best of the authors’ knowledge, this study makes several important contributions. First, it is the first study to analyze the determinants of life expectancy in the top countries with the highest life expectancy. Second, the study integrates environmental, economic, social, and institutional factors, whereas previous studies, such as Li et al. (72), focused primarily on economic and social determinants. The factors considered in this study include CO₂ emissions (environmental), GDP, health expenditure, and research & development (economic), education and individual internet use (social), and rule of law and government effectiveness (institutional). Third, the inclusion of R&D as a determinant is a novel contribution, as it has largely been ignored in prior literature. Fourth, this study uniquely combines traditional econometric techniques, including PMG, Fixed Effects, and FMOLS, with advanced machine learning methods such as Gradient Boosting and Random Forest. In contrast, previous research has generally relied only on conventional methods. Finally, the study provides policy recommendations aimed at enhancing Sustainable Development Goals (SDGs), particularly to improve life expectancy through environmental management, economic growth, social development, and institutional strengthening.

## 2 Literature review

### 2.1 Empirical review

Shaw et al. (73) analyzed the determinants of LEX using OECD health data for developed countries. The authors employed an aggregate LEX production function, controlling for the influence of age distribution to address omitted-variable bias. Findings indicated that pharmaceutical consumption positively impacted LEX at middle and advanced ages, with the effect size depending on the age composition of the population. Specifically, doubling annual pharmaceutical expenditure increased male LEX at age 40 by approximately one year and female LEX at age 65 by slightly less than one year. Lifestyle factors also played a role, as reducing tobacco consumption by two cigarettes per day or increasing daily fruit and vegetable consumption by one-third of a pound could increase LEX by about one year for females aged 40. Kabir (74) examined the socioeconomic determinants of LEX in 91 developing countries using data from an unspecified period. The study applied multiple regression and disaggregated probit regression frameworks, separating countries into low, medium, and high LEX groups. The analysis revealed that most explanatory variables, including per capita income, education, health expenditure, access to safe water, and urbanization, were statistically insignificant in explaining LEX variations across the Sample. The author concluded that these socioeconomic indicators cannot always be considered decisive in determining LEX in developing countries. Policy recommendations included enhancing the availability of physicians, reducing adult illiteracy, and addressing undernourishment to improve overall LEX.

Lin et al. (75) analyzed the political and socioeconomic determinants of LEX in 119 less developed countries (LDCs) from 1970 to 2004. Using linear mixed models with lag effects of up to 10 years, the study examined GDP per capita, literacy rates, undernourishment, and political regime. Results indicated that all four factors contributed to increases in LEX, with political regime initially having the least impact but becoming significant from the third year onwards. The influence of economic, educational, and nutritional factors declined over time, while the long-term positive role of democracy strengthened, contributing to up to 98% of LEX gains in some lag periods.

Bayati et al. (76) estimated a health production function for 21 Eastern Mediterranean region from 1995 to 2007 using panel data and fixed effects based on the Hausman test. The study identified per capita income, education index, food availability, urbanization, and employment ratio as significant positive determinants of LEX. A gender-specific analysis revealed that the elasticity of LEX concerning employment was higher for males than for females. The authors recommended that policymakers focus on economic growth and employment generation to improve public health outcomes. Monsef and Mehrjardi (77) investigated economic, social, and environmental determinants of LEX in 136 countries from 2002 to 2010. Using panel data regression techniques, the authors found that unemployment and inflation were the main economic factors negatively affecting LEX, while gross capital formation and gross national income had positive effects. Urbanization emerged as the primary socio-environmental factor influencing mortality. Based on these findings, the study recommended economic stabilization policies, job creation programs, and improved urban living conditions as means to extend LEX. Sede and Ohemeng (78) examined the socioeconomic determinants of LEX in Nigeria from 1980 to 2011, focusing on the country’s progress toward the Millennium Development Goal of achieving 70 years of LEX by 2020. Using Vector Autoregression (VAR) and Vector Error Correction Model (VECM) frameworks to address endogeneity, the study included variables such as secondary school enrollment, government health expenditure, per capita income, unemployment rate, and the Naira foreign exchange rate. Results showed that traditional determinants like income, education, and health expenditure were insignificant in Nigeria’s case. In contrast, exchange rate stability, reduced unemployment, and improved quality of government health spending were critical for enhancing LEX.

Gilligan and Skrepnek (79) examined socioeconomic and health expenditure determinants of LEX in 21 Eastern Mediterranean Region (EMR) countries from 1995 to 2010. The authors used WHO and World Bank data, applying cluster analysis to group countries by development level and employing multilevel mixed-effects linear models with distributed lags. Results showed that GDP, vaccination rates, and urbanization were significant positive predictors of LEX overall. The predictors varied by cluster: in less developed nations, physician density and vaccination rates mattered most; in more developed clusters, GDP, literacy, and health expenditures were key determinants. Hassan et al. (80) analyzed social environment determinants of LEX in 108 developing countries from 2006 to 2010 using panel data methods. The results showed that GDP, education index, health expenditure, improved water coverage, and sanitation facilities all had positive effects on LEX, with education and GDP being the most significant predictors. Causality analysis revealed no short-run causality but demonstrated long-run unidirectional causality from these determinants to LEX, and bidirectional causality between income and LEX.

Rahman et al. (81) examined the determinants of LEX in the 31 most polluted countries from 2000 to 2017, focusing on environmental degradation by using the Panel Corrected Standard Errors. The study confirmed a positive relationship between economic growth and LEX, while CO2E has a negative effect on LEX. Moreover, health expenditure, access to clean water, and improved sanitation increased it. Azam et al. (82) investigated environmental degradation and socioeconomic factors affecting LEX in Pakistan from 1975 to 2020 using ARDL bounds testing and Johansen cointegration techniques. The study found that death rate, food production index, inflation, and CO2E have negatively affected LEX, while income, health expenditure, urbanization, birth rate, and education have had positive impacts. Ahmad et al. (83) analyzed the impact of urbanization and income inequality on male and female LEX in six South Asian countries from 1997 to 2021 using random effects models. Results showed that urbanization and income inequality significantly reduced LEX for both genders, while health expenditure increased it. Interaction analysis indicated that health expenditure mitigated the adverse effects of urbanization, though with a small effect size, highlighting the importance of public health investments in urban areas. Uddin et al. (84) explored how institutional quality, financial development, and environmental pressures shape LEX across SAARC from 2002 to 2020. They reported that stronger institutions, financial sector progress, and greater health spending support improvements in LEX. Conversely, environmental stressors such as higher CO₂ emissions, larger ecological footprints, demographic pressures, and adverse birth–mortality dynamics were linked to reductions in LEX.

### 2.2 Theoretical review

Uddin et al. (84) extended Grossman’s health production function by incorporating additional variables, including environmental degradation, financial development, and institutional factors. Their findings indicate that environmental degradation negatively affects LEX, whereas financial development and institutional quality exert positive influences on LEX. Environmental degradation can disrupt food supply and reduce water quality, which in turn increases mortality risks, especially for infants, the older population, and disadvantaged groups from lower-income communities (81). Moreover, poor air quality has been shown to strongly affect the life expectancy of older population individuals, who are less capable of adapting to environmental stress because of pre-existing health conditions (85, 86). Information and communication technologies, along with developments in biomedical engineering, drug design, disease management, and medical science, present substantial chances to enhance health outcomes through programs like more efficient business procedures and better and more affordable patient diagnosis and treatment. These intermediate results are likely to lead to a healthcare system that is more efficient, less expensive, and of excellent quality (87–89). Education affects life expectancy through various direct and indirect channels. Higher education levels are associated with increased health awareness, prevention, protection, and promotion of public health, as well as better knowledge of healthy lifestyle habits such as nutrition and physical activity (71). Education helps people develop effective human agency, which in turn encourages healthy lifestyles.

## 3 Methodology

### 3.1 Model construction and data

This study examines the determinants of life expectancy in the world’s top 20 countries with the highest longevity. The empirical model applied in the analysis is derived from:


$$\begin{array}{l} {\mathrm{LEX}}_{\mathrm{it}}=\gamma_{0}+\gamma_{1}C02E_{\mathrm{it}}+\gamma_{2}{\mathrm{GDP}}_{\mathrm{it}}+\gamma_{3}{\mathrm{HE}}_{\mathrm{it}}+\gamma_{4}{\mathrm{EDU}}_{\mathrm{it}}\\ +\gamma_{5}{\mathrm{IUI}}_{\mathrm{it}}+\gamma_{6}{\mathrm{ROL}}_{\mathrm{it}}+\gamma_{7}{\mathrm{GE}}_{\mathrm{it}}\gamma_{8}+R\&D_{\mathrm{it}}+u_{t} \end{array}$$ (1)

Where in Equation 1, LEX, CO2E, GDP, HE, EDU, IUI, ROL, GE and R&D represent the life expectancy (at birth, total, years), Carbon dioxide emissions (CO2E) (per capita, CO2e/capita), Gross domestic product (per capita, constant 2015 US$), health expenditure [(% of GDP), education (School enrollment, secondary, % gross), Individuals using the Internet (% of population), Rule of Law (Estimate), Government Effectiveness (Estimate), and Research and development expenditure (% of GDP) respectively]. The data has been obtained from the world bank and UNDP. This study utilized the full dataset covering the period from 2001 to 2023 for the chosen regression analysis and transformed variables into their natural logarithmic form. The benefits of this transformation in that, it addresses heteroskedasticity issues and facilitate interpretation, the variables LEX, CO₂E, and GDP were transformed, while the remaining variables were already expressed as percentages, except for ROL and GE, which retained their original values due to the presence of negative observations. For the machine learning analysis, this study first normalized the data using the StandardScaler method. This process adjusts each feature to have a mean of zero and a standard deviation of one. Normalizing the data helps reduce the influence of outliers, which can distort the analysis and decrease model accuracy. In this study, the dataset was randomly split into two parts: 70% for training and 30% for testing. The training data were used to help the Random Forest and Gradient Boosting models learn the patterns and relationships within the variables, while the testing data were used to evaluate the models’ performance on unseen data. Random splitting offers several advantages. It ensures that both subsets represent the overall data distribution, reducing the risk of bias that could occur if the split followed a specific order or pattern. This approach helps improve the generalizability of the model, making its predictions more reliable when applied to new datasets. It also prevents overfitting, as the model is tested on data it has not previously encountered. This study used the ranger package for Random Forest and the xgboost package for Gradient Boosting. Both are efficient and widely used R libraries for accurate and scalable machine learning analysis.

### 3.2 Methodology

#### 3.2.1 Cross sectional dependency

Cross-sectional dependency (CSD) refers to the correlation of error terms across panel units, often arising from common shocks, spillover effects, or unobserved global factors (90). Ignoring CSD can lead to biased and inconsistent estimators in panel regressions. One common method to detect cross-sectional dependence (CSD) is by applying the Lagrange Multiplier (LM) test, as shown in Equation 2, and the Cross-sectional Dependence (CD) test, as shown in Equation 3:


$$\mathrm{LM}=\sum_{i=1}^{N-1}\sum_{j=i+1}^{N}T{{\hat{\omega }}^{2}}_{\mathrm{ij}}$$ (2)

$$\mathrm{CD}=\sqrt{\frac{2}{N(N-1)}}\sum_{i=1}^{N-1}\sum_{j=i+1}^{N}{\hat{\omega }}_{\mathrm{ij}}$$ (3)

where ${\overset{ˆ}{\omega }}_{\mathrm{ij}}$ is the pairwise correlation coefficient of residuals between units i and j . The null hypothesis is $H_{0}:\omega_{\mathrm{ij}}=0$ for all i≠j , implying no CSD. Under $H_{0,}\mathrm{CD}∼N(0,1)$ . Rejecting $H_{0}$ indicates significant cross-sectional correlation.

#### 3.2.2 Panel unit root test

The Levin, Lin, and Chu (LLC) test, proposed by Levin et al. (91), examines the null hypothesis that all panels contain a unit root against the alternative that all are stationary. The method assumes a common autoregressive coefficient across cross-sections, allowing for individual intercepts and time trends. LLC corrects for serial correlation and heteroskedasticity using a bias-adjusted t-statistic, but it requires the variables to have the same order of integration In contrast, the Cross-sectionally Augmented IPS (CIPS) test, developed by Pesaran (92), extends the Im et al. (93) approach by accounting for cross-sectional dependence, which is common in macroeconomic and financial panel datasets. CIPS augments the standard ADF regressions with cross-section averages of lagged levels and first differences of the series. The test allows for heterogeneity in autoregressive coefficients and is robust to cross-sectional correlation.

#### 3.2.3 PMG estimator and FMOLS

This study applies the Pooled Mean Group (PMG) estimator developed by Pesaran et al. (94). Earlier studies, including Phillips and Hansen (95) and Johansen (96), noted that long-run relationships typically require variables to share the same integration order. Pesaran et al. (112), building on Pesaran and Shin (97), showed that the ARDL model remains valid when the regressors include a mix of I(0) and I(1) variables. This makes the PMG estimator appropriate for this analysis because it addresses potential endogeneity through the use of lagged dependent and explanatory variables. In this study, the panel ARDL model of order $\left(p,q_{1},q_{2},\ldots ,q_{k}\right)$ proposed by Pesaran et al. (94) is specified in Equation 4:


$$Y_{\mathrm{it}}=\sum_{j=1}^{p}\;\alpha_{i,j}Y_{i,t-j}+\sum_{m=1}^{k}\;\sum_{j=0}^{q_{m}}\;\beta_{i,m,j}X_{m,i,t-j}+\mu_{i}+\varepsilon_{\mathrm{it}}$$ (4)

Here, i denotes the cross-sectional unit (i=1,2,…,N),t represents the time period (t=1,2,…,T) , and N=20 in this application covering 2001–2023. The dependent variable $Y_{\mathrm{it}}$ corresponds to the outcome of interest (LEX), while $X_{m,\mathrm{it}}(m=1,2,\ldots ,k)$ denotes the k explanatory variables. $\mu_{i}$ captures unobserved country-specific effects, $\alpha_{i,j}$ are the coefficients on the lagged dependent variable, $\beta_{i,m,j}$ are the coefficients on the lagged explanatory variables, and $\varepsilon_{\mathrm{it}}$ is the idiosyncratic error term. For consistent estimation, T must be sufficiently large to allow each country’s equation to be estimated independently (98). The error correction representation of the model can be expressed as:


$$\begin{array}{l} \Delta Y_{\mathrm{it}}=\lambda_{i}(Y_{i,t-1}-\theta_{i}^{\prime }X_{i,t})+\sum_{j=1}^{p-1}\phi_{i,j}\Delta Y_{i,t-j}\\ +\sum_{m=1}^{k}\sum_{j=0}^{q_{m}-1}\psi_{i,m,j}\Delta X_{m,i,t-j}+\mu_{i}+\varepsilon_{\mathrm{it}} \end{array}$$ (5)

Where in Equation 5, the $\lambda_{i}$ , $\theta_{i}$ , $\phi_{i,j}$ and $\psi_{i,m,j}$ represent the speed of adjustment toward the long-run equilibrium, vector of long-run coefficients, short-run coefficients of the lagged dependent variable and short-run coefficients of the lagged explanatory variables, respectively.

The Fully Modified Ordinary Least Squares (FMOLS) procedure, first proposed by Phillips and Hansen (95), is designed to yield efficient estimates in cointegrating regressions. For panel settings, the heterogeneous FMOLS method developed by Pedroni (99) is applied, as it effectively handles both endogeneity bias and serial correlation in the regressors. According to Hamit-Haggar (100), this framework, which accommodates heterogeneity in cointegration relationships, is well-suited for panel data analysis. Kao and Chiang (101) further emphasize that FMOLS mitigates the bias found in conventional least squares estimators when explanatory variables suffer from endogeneity and autocorrelation. As noted by Pedroni (99), the method is a non-parametric approach that adjusts for serial correlation, and when coefficients are stationary with variables being cointegrated, it reduces distortions in estimation outcomes (98).

If the coefficient γ in model (1) is estimated using FMOLS, the expression can be represented in Equation 6:


$$\begin{array}{l} {\hat{\gamma }}_{\mathrm{mn}}^{*}-\gamma =\sum_{i=1}^{M}\kappa_{22,i}^{-2}\sum_{t=1}^{N}(x_{\mathrm{it}}-{\bar{x}}_{\mathrm{it}})\\ -\sum_{i=1}^{M}\kappa_{11,i}^{-1}\kappa_{22,i}^{-1}(\sum_{t=1}^{N}(x_{\mathrm{it}}-{\bar{x}}_{\mathrm{it}})u_{\mathrm{it}}^{*}-T{\hat{\gamma }}_{i})\prime \end{array}$$ (6)

where:


$$u_{\mathrm{it}}^{*}=u_{\mathrm{it}}-\frac{{\hat{L}}_{21,i}}{{\hat{L}}_{22,i}}\Delta x_{\mathrm{it}},{\hat{\gamma }}_{i}={\hat{\hbar }}_{21,i}{\hat{\Omega }}_{21,i}^{\left(0\right)}-\frac{{\hat{L}}_{21,i}}{{\hat{L}}_{22,i}}({\hat{\sigma }}_{22,i}^{2}+{\hat{\Omega }}_{22,i}^{\left(0\right)})$$

Here, $x_{\mathrm{it}}$ denotes the regressor, ${\bar{x}}_{\mathrm{it}}$ is its mean-adjusted value, $u_{\mathrm{it}}^{*}$ is the bias-corrected residual, $\Delta x_{\mathrm{it}}$ represents the first difference of $x_{\mathrm{it}}$ , and ${\hat{L}}_{21,i},{\hat{L}}_{22,i},\kappa_{11,i}$ , and $\kappa_{22,i}$ are kernel-based long-run variance and covariance estimates.

#### 3.2.4 Random forest

Random Forest (RF) is an advanced ensemble learning framework designed to integrate the predictive strengths of numerous decision trees, each cultivated from unique bootstrap samples of the original dataset. By fostering diversity among the trees and subsequently averaging their outputs, the method substantially curtails model variance, delivering superior predictive accuracy and a pronounced safeguard against overfitting. Its prediction process is mathematically is specified in Equation 7:


$$\hat{Y}=\frac{1}{H}\sum_{h=1}^{H}f_{h}(X)$$ (7)

Where $\hat{Y}$ denotes the ensemble’s prediction, *H* is the number of constituent trees, $f_{h}$ is the prediction function of the hth tree, and *X* encapsulates the vector of explanatory variables. This architecture proves especially potent in domains marked by high-dimensional feature spaces and intricate inter-variable dependencies, where it consistently delivers resilient, generalizable, and analytically sound models capable of capturing the subtle complexities inherent in real-world data (102).

#### 3.2.5 Gradient boosting

Gradient Boosting (GB), developed by Friedman (103), is an ensemble machine learning technique designed to improve prediction accuracy by combining several weak learners. It builds regression trees in an iterative process, splitting the data into smaller subsets to minimize residual errors. The model starts with all observations in a single group, then repeatedly partitions them based on the predictor that most effectively reduces the error, measured using Friedman’s Mean Squared Error (MSE). Through this approach, GB enhances the model’s accuracy and reliability in estimating continuous outcomes (104). GB is a powerful ensemble learning methodology that constructs predictive models in a sequential, stage-wise manner, typically employing decision trees as its base learners. Unlike methods that build models independently, Gradient Boosting strategically focuses each new model on the residual errors of its predecessors, thereby iteratively refining predictive performance through the minimization of a specified loss function. The process can be mathematically is expressed in Equation 8:


$$F(X)=F_{0}(X)+\sum_{m=1}^{M}\;\gamma_{m}h_{m}(X)$$ (8)

Here, $F_{0}(X)$ denotes the initial model, serving as the foundation of the boosting process; M represents the total number of boosting iterations or stages; $h_{m}(X)$ is the prediction of the $m^{\mathrm{th}}$ base learner-often a shallow decision tree-trained on the residuals from the preceding stage; and $\gamma_{m}$ is the weight assigned to that learner’s contribution (102).

This approach is particularly adept at capturing intricate, nonlinear relationships between predictors and the target variable, offering adaptive flexibility and high predictive precision. By integrating both regression techniques and machine learning capabilities, GB not only elucidates the relative importance of each predictor but also produces models that are both resilient and analytically rigorous-enabling more accurate forecasts and deeper strategic insights, such as in the analysis of life expectancy dynamics.

## 4 Results and discussions

### 4.1 Results

Table 1 shows the descriptive statistics where the mean value of LEX, CO2E, GD, HE, EDU, IUI, ROL, GE and RD are 81.25, 10.90, 47171.05, 8.37, 110.75, 75.05, 1.42, 1.45 and 2.10, respectively. The standard deviation of LEX, CO2E, GD, HE, EDU, IUI, ROL, GE and R&D are 1.67, 9.36, 21855.66, 2.35, 15.72, 21.12, 0.44 0.46 and 1.06, respectively. The correlation matrix shows in Table 2, LEX is strongly correlated to IUI (0.64) and moderately to R&D (0.26), but negatively to CO2E (−0.15). GDP has strong positive links with ROL (0.56) and GE (0.52), while HE is negatively related to CO2E (−0.45). Table 3 shows the estimates of Variance Inflation Factor (VIF) It measures how much the variance of a regression coefficient is inflated due to multicollinearity among independent variables. A VIF of 1 means no correlation with other predictors, while higher values indicate stronger collinearity. The mean VIF value is 2.57, which is well below the common threshold of 05, indicating no serious multicollinearity issue in the model with LEX as the dependent variable. This suggests that the independent variables are not highly linearly related overall, and the regression estimates should remain stable.

**Table 1:** Descriptive statistics.

Variable	Mean	Std. Dev.	Min	Max
LEX	81.25	1.67	75.49	84.56
CO2E	10.90	9.36	3.00	53.60
GDP	47171.05	21855.66	16920.73	112417.88
HE	8.37	2.35	1.60	13.02
EDU	110.75	15.72	87.48	164.08
IUI	75.05	21.12	6.17	99.80
ROL	1.42	0.44	0.21	2.02
GE	1.45	0.46	0.19	2.47
R&D	2.10	1.06	0.24	6.02

**Table 2:** Correlation matrix.

Variables	LEX	CO2E	GDP	HE	EDU	IUI	ROL	GE	R&D
LEX	1								
CO2E	−0.15	1							
GDP	0.18	0.42	1						
HE	0.19	−0.45	−0.25	1					
EDU	−0.02	−0.14	0.05	0.32	1				
IUI	0.64	0.14	0.45	0.13	0.19	1			
ROL	−0.01	0.11	0.56	0.15	0.22	0.42	1		
GE	0.01	0.13	0.52	0.04	0.21	0.37	0.59	1	
R&D	0.26	−0.10	−0.07	0.06	−0.08	0.27	0.01	0.17	1

**Table 3:** VIF.

Variables	VIF	1/VIF
ROL	4.23	0.14
GE	6	0.17
GDP	2.5	0.4
IUI	1.9	0.53
HE	1.72	0.58
CO2E	1.54	0.65
R&D	1.41	0.71
EDU	1.26	0.8
Mean VIF	2.57	

Table 4 shows the results of CSD test, The Breusch–Pagan LM (616.270, *p* = 0.000), Pesaran scaled LM (21.867, *p* = 0.000), and Bias-corrected scaled LM (21.367, *p* = 0.000) all reject the null hypothesis of cross-sectional independence. The Pesaran CD test (13.304, *p* = 0.000) confirms significant correlations across cross-sections. These four statisitcs confirms that thre is CSD.

**Table 4:** CSD test.

Test	Statistic	Prob.
Breusch-Pagan LM	616.270	0.000
Pesaran scaled LM	21.867	0.000
Bias-corrected scaled LM	21.367	0.000
Pesaran CD	13.304	0.000

Thus, panel estimations should account for cross-sectional dependence to avoid biased standard errors.

Table 5 presents the results of the LLC and CIPS unit root tests. In the LLC test, at level, LEX, GDP, IUI, and GE are stationary, while in the CIPS test, at level, LEX, EDU, IUI, and GE are stationary. After the first difference, both tests confirm that all variables are stationary at first differences.

**Table 5:** Unit root test.

Variables	LLC		CIPS	
	I(0)	I(1)	I(0)	I(1)
LEX	−7.25^*^	−3.92^*^	−2.91^*^	−3.73^*^
CO2E	2.88	−6.31^*^	−1.86	−5.63^*^
GDP	−1.80^**^	−14.12^*^	−1.80	−10.74^*^
HE	−0.76	−10.57^*^	−1.57	−3.35^*^
EDU	−1.23	−6.45^*^	−4.53^*^	−4.90^*^
IUI	−11.83^*^	−5.61^*^	−2.74^*^	−3.53^*^
ROL	0.05	−6.79^*^	−1.67	−4.82^*^
GE	−1.57^***^	−6.26^*^	−2.67^*^	−6.02^*^
R&D	−0.32	−4.56^*^	−1.86	−5.38^*^

Table 6 shows the Kao residual cointegration test, The Kao test statistic (−2.3659, *p* = 0.009) is significant, rejecting the null hypothesis of no cointegration. This implies a long-run equilibrium relationship exists among the variables in the panel. The residual variance (0.1274) indicates the average variation of the residuals. The HAC variance (0.0828) provides a robust estimate, confirming the stability of the long-run relationship.

**Table 6:** Kao residual cointegration test.

Test	t-statistic	Prob.
ADF	−2.3659	0.009
Residual variance	0.127436	
HAC variance	0.082823	

Table 7 shows the PMG estimates, The PMG long-run results show that CO₂ emissions (−0.050, *p* < 0.01) significantly reduce life expectancy, while GDP (0.804, *p* < 0.01), health expenditure (0.634, *p* < 0.05), education (0.372, *p* < 0.01), rule of law (0.240, *p* < 0.05), government effectiveness (0.606, *p* < 0.01), and R&D expenditure (0.155, *p* < 0.01) significantly enhance life expectancy, highlighting the crucial roles of economic growth, social investment, and institutions. In contrast, individual internet use (0.890, *p* > 0.1) is statistically insignificant in the long run, suggesting that its positive impact emerges only gradually. In the short run, CO₂ emissions (−0.104, *p* < 0.01) remain detrimental to LEX, whereas GDP (0.534, *p* < 0.01), health expenditure (0.164, *p* < 0.01), education (0.021, *p* < 0.01), internet use (0.049, *p* < 0.01), and R&D (0.467, *p* < 0.01) significantly improve life expectancy. The rule of law (0.563, *p* = 0.061) shows a 10% significant effect, while government effectiveness (0.150, *p* > 0.1) is insignificant in the short run. The negative and significant ECM(−1) coefficient (−0.246, *p* < 0.01) confirms the presence of a long-run equilibrium, with about 24.6% of disequilibrium corrected each period, ensuring convergence toward stability.

**Table 7:** PMG estimates.

Variable	Long run			Short run		
	Coefficient	Std. Error	Prob.	Coefficient	Std. Error	Prob.
CO2E	−0.050	0.003	0.000	−0.104	0.025	0.000
GDP	0.804	0.033	0.000	0.534	0.019	0.000
HE	0.634	0.291	0.043	0.164	0.046	0.000
EDU	0.372	0.026	0.000	0.021	0.005	0.000
IUI	0.890	0.832	0.384	0.049	0.003	0.000
ROL	0.240	0.089	0.023	0.563	0.299	0.061
GE	0.606	0.072	0.000	0.150	0.215	0.487
R&D	0.155	0.021	0.000	0.467	0.112	0.000
ECM(−1)				−0.246	0.030	0.000

Table 8 shows the robustness analysis, the results of the Fixed Effects and FMOLS estimations are consistent with the ARDL long-run findings. CO2E exert a negative effect on LEX, while GDP, health expenditure, education, individual internet use, rule of law, government effectiveness, and R&D all show positive effects.

**Table 8:** Robustness analysis.

Variable	Fixed effect			FMOLS		
	Coefficient	Std. error	Prob.	Coefficient	Std. error	Prob.
CO2E	−0.118	0.023	0.000	−0.121	0.034	0.001
EDU	0.022	0.005	0.000	0.028	0.007	0.000
GDP	0.000	0.000	0.000	0.000	0.000	0.014
GE	0.169	0.059	0.024	0.975	0.297	0.000
HE	0.585	0.045	0.000	0.760	0.066	0.000
IUI	0.039	0.003	0.000	0.037	0.005	0.000
ROL	0.579	0.278	0.038	0.407	0.407	0.319
R&D	0.493	0.104	0.000	0.633	0.151	0.000
C	71.035	0.777	0.000			

### 4.2 Machine learning results

**Figure 2:**
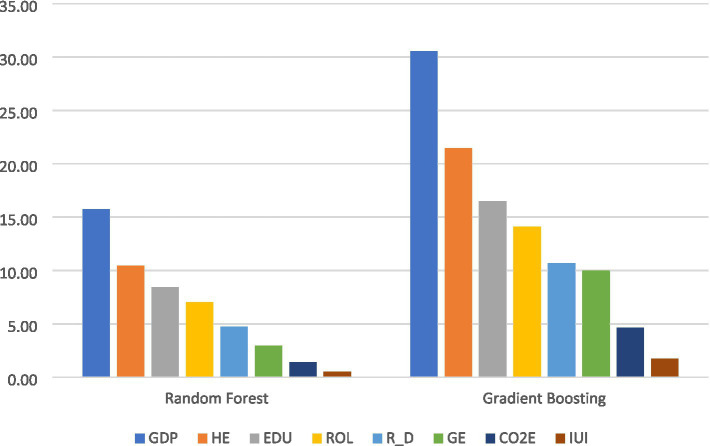
Feature importance coefficient for machine learning models.

Figure 2 illustrates the comparative importance of predictors as estimated by two ensemble-based machine learning techniques: Random Forest and Gradient Boosting. Both approaches assess the extent to which each variable contributes to explaining variations in the dependent variable, life expectancy, yet they rely on distinct algorithmic frameworks and importance metrics to quantify these contributions. GDP emerges as the most influential predictor in both Gradient Boosting and Random Forest models, with importance scores of 30.54 and 15.74, respectively, underscoring its dominant role in shaping LEX. Health expenditure follows as the second most significant factor, recording scores of 21.46 and 10.47, respectively. Education holds the third position in predictive influence, with scores of 16.47 and 8.43, reflecting its substantial, though slightly lesser, impact compared to GDP and HE. Rule of Law exhibits moderate importance, with scores of 14.10 in Gradient Boosting and 7.01 in Random Forest. Research and Development contributes less prominently, scoring 10.66 and 4.73, respectively. Government Effectiveness (GE) shows relatively lower influence, with importance values of 10.00 and 2.93, respectively. CO₂ emissions rank near the bottom, with scores of 4.64 and 1.40, while the Internet Usage is consistently the least important predictor, with scores of 1.74 and 0.49, respectively. Both models present a consistent ranking of predictor importance: GDP > HE > EDU > ROL > R & D > GE > CO2E > IUI. Notably, Gradient Boosting assigns higher and more differentiated numerical importance values, suggesting a sharper distinction between variable contributions, whereas Random Forest outputs are more compressed and normalized. In both methodologies, GDP and HE are the most critical drivers of LEX, while CO2E and IUI exert minimal influence, indicating their limited relevance across model frameworks.

For the prediction of LEX, Gradient Boosting outperformed Random Forest across most evaluation metrics. Gradient Boosting achieved a lower MAE (0.7346 vs. 1.5784) and RMSE (1.734 vs. 2.743), alongside a higher 𝑅^2^ value (0.9854 vs. 0.9633), indicating superior accuracy and model fit. Although Random Forest recorded a slightly lower MAPE (55.84 vs. 60.73), the overall results confirm Gradient Boosting’s stronger predictive capability in modeling life expectancy.

### 4.3 Discussions

The finding shows that the CO2E has an adverse effect on LEX, which is why the negative sign occurs; CO2E reduces LEX because it worsens air quality and intensifies environmental stress. Polluted air raises the incidence of respiratory and cardiovascular diseases, leading to higher mortality. Moreover, CO₂-driven climate change increases heatwaves, natural disasters, and disease spread, all of which shorten human lifespan. The finding is consistent with the findings of Azam et al. (82) and Uddin et al. (84). Azam et al. (82) analyzed the CO2E and LEX in Pakistan, and they found that CO2E reduces the LEX. Uddin et al. (84) reported that CO2E reduces the LEX in SAARC economies.

The education coefficient has a positive effect on LEX; thus, education enhances LEX by encouraging healthier lifestyles, improving access to healthcare, and strengthening health literacy. Individuals with higher education are better equipped to recognize health risks, practice preventive care, and make informed choices about diet, exercise, and medical treatment. Furthermore, education creates economic opportunities that reduce poverty-related health challenges and increase access to clean water, sanitation, and nutritious food (35, 36).

The GDP and health expenditure have a positive effect on LEX. GDP is positively associated with LEX, higher GDP boosts LEX by increasing national income, which allows greater investment in healthcare, education, and infrastructure. It improves access to quality medical services and advanced technologies that reduce mortality. Rising GDP also elevates living standards, providing better housing, sanitation, and nutrition. Moreover, stronger economic growth creates the conditions for healthier and longer lives. The finding is consistent with the findings of Amin et al. (6) and Țarcă et al. (105), and they found that economic growth and health expenditure have a positive effect on LEX in ASEAN-5 countries and Eastern Europe.

The individual using the internet has a positive effect on LEX; thus, Individual use of the internet enhances LEX by improving access to health information and raising awareness about preventive care. It facilitates better communication with healthcare providers and access to online medical services. Moreover, internet use promotes health literacy and informed decision-making, leading to healthier lifestyles and longer lives. Lee and Kim (106) reported that the internet environment has a positive effect on LEX in Asia. Kim and Kim (107) reported that the internet enhances the LEX in 178 countries. Pu et al. (108) analyzed the internet use, which significantly and positively impacts LEX in 182 countries.

The rule of law has a positive effect on LEX; thus, a stronger rule of law enhances LEX by ensuring equal access to justice and protection of fundamental rights. It promotes fair distribution of resources and reduces corruption, allowing better delivery of healthcare and social services. Effective legal systems also enforce health, safety, and environmental regulations, safeguarding public wellbeing. Moreover, the rule of law creates a stable and secure environment that supports healthier and longer lives. This finding is consistent with the findings of Pinzon-Rondon et al. (109), who found that the rule of law significantly enhances health outcomes. The R&D has a positive effect on LEX; thus, R&D enhances LEX by driving medical innovations such as new treatments, vaccines, and diagnostic technologies. It improves healthcare efficiency and accessibility, reducing mortality from preventable diseases. Moreover, R&D also supports technological and pharmaceutical advancements that contribute to longer and healthier lives. Muradov et al. (110) reported that medical innovation enhances the LEX in the USA.

## 5 Conclusion

The primary objective of this empirical study is to examine the determinants of LEX in the top 20 countries with the highest life expectancies during the period 2001–2023. The analysis integrates both traditional econometric techniques namely PMG, Fixed Effects, and FMOLS estimators and advanced machine learning approaches such as Gradient Boosting and Random Forest. The regression results reveal that CO₂ emissions exert a negative impact on LEX, whereas GDP, health expenditure, education, and individual internet use, rule of law, government effectiveness, and R&D positively influence LEX. Meanwhile, the machine learning models highlight GDP and health expenditure as the most critical drivers of LEX, while CO₂ emissions and internet use display relatively minor effects, suggesting limited significance across model frameworks. For predictive performance, Gradient Boosting consistently outperforms Random Forest, achieving lower MAE and RMSE alongside a higher R^2^.

This study has several policy implications to enhance the LEX. First, Governments should reduce reliance on fossil fuels by expanding investment in renewable energy sources such as solar, wind, and hydropower, thereby lowering CO2E and improving public health. Second, enforcing stricter emission standards for industries, vehicles, and urban development can curb air pollution, reduce disease burden, and enhance LEX. Third, developing eco-friendly public transport, green spaces, and sustainable waste management systems will improve air quality, mitigate CO2E, and create healthier living environments that support longer lifespans. Fourth, ensure strict implementation of health, environmental, and labor laws so citizens have safer working conditions, cleaner environments, and better access to justice, which directly improves health outcomes. Fifth, Curb corruption and improve efficiency in public service delivery, especially in healthcare and education, so that resources reach vulnerable populations and contribute to longer, healthier lives. Six, build effective institutions that can design and implement evidence-based health policies, ensure equitable healthcare access, and improve emergency response systems, thereby extending LEX. Seventh, Governments should allocate more funding to medical and healthcare research to develop new treatments, vaccines, and diagnostic tools that directly improve population health and longevity. Eight, Encourage private sector and university collaborations to promote technological advancements in medical devices, telemedicine, and health monitoring systems, enhancing the quality and accessibility of healthcare. Ninth, implement programs that train healthcare professionals and disseminate research findings nationwide, ensuring that R&D innovations translate into practical improvements in health services and LEX.

In conclusion, this study acknowledges several limitations that can guide future research. First, the analysis focused exclusively on the top 20 countries with the highest LEX, which may limit the generalizability of the findings. Future studies should consider a broader set of countries, including developed, emerging, and developing nations, to capture more diverse socio-economic and institutional contexts. Second, this study examined only a limited set of variables as determinants of LEX, and additional factors such as environmental quality, healthcare infrastructure, and social policies could be incorporated in subsequent research. Third, while the study employed the traditional PMG estimator, it did not consider alternative approaches such as the CS-ARDL model, which may provide additional insights into heterogeneous dynamics across countries. Finally, in terms of machine learning methods, the study relied solely on Random Forest and Gradient Boosting techniques; future research should explore other advanced ML approaches, such as XGBoost, LightGBM, or deep learning models, to improve predictive accuracy and robustness.

## Data Availability

The raw data supporting the conclusions of this article will be made available by the authors, without undue reservation.
