# Correction: Determinants of life expectancy in high longevity countries: evidence from machine learning

**DOI:** 10.3389/fpubh.2025.1764102

**Published:** 2026-01-06

**Authors:** Frontiers Production Office

**Affiliations:** Frontiers Media SA, Lausanne, Switzerland

**Keywords:** economic, social, institutional, life expectancy, machine leaning

Author Hong Zhang was erroneously assigned to affiliation “1 School of Business, Jinggangshan University, Ji'an, China”. This affiliation has now been removed for author Hong Zhang

The order of corresponding authors in the author list of the published paper was erroneously given as:

Xiaolan Zhang and Ruitao Li.

The correct order of the corresponding authors list reads:

Ruitao Li and Xialan Zhang.

The original version of this article has been updated.

